# Bacterial and fungal chitinase *chiJ* orthologs evolve under different selective constraints following horizontal gene transfer

**DOI:** 10.1186/1756-0500-5-581

**Published:** 2012-10-24

**Authors:** Wimal Ubhayasekera, Magnus Karlsson

**Affiliations:** 1 Department of Medical Biochemistry and Microbiology (IMBIM), Biomedical Center, Uppsala University, Box 582, SE-75123, Uppsala, Sweden; 2 Uppsala BioCenter, Department of Forest Mycology and Plant Pathology, Swedish University of Agricultural Sciences, Box 7026, SE-75007, Uppsala, Sweden

**Keywords:** Chitinase, Functional divergence, Homology modelling, Horizontal gene transfer, *Hypocrea*, Protein evolution, *Streptomyces*, *Trichoderma*

## Abstract

**Background:**

Certain bacteria from the genus *Streptomyces* are currently used as biological control agents against plant pathogenic fungi. Hydrolytic enzymes that degrade fungal cell wall components, such as chitinases, are suggested as one possible mechanism in biocontrol interactions. Adaptive evolution of chitinases are previously reported for plant chitinases involved in defence against fungal pathogens, and in fungal chitinases involved in fungal-fungal interactions. In this study we investigated the molecular evolution of chitinase *chiJ* in the bacterial genus *Streptomyces*. In addition, as *chiJ* orthologs are previously reported in certain fungal species as a result from horizontal gene transfer, we conducted a comparative study of differences in evolutionary patterns between bacterial and fungal taxa.

**Findings:**

*ChiJ* contained three sites evolving under strong positive selection and four groups of co-evolving sites. Regions of high amino acid diversity were predicted to be surface-exposed and associated with coil regions that connect certain α-helices and β-strands in the family 18 chitinase TIM barrel structure, but not associated with the catalytic cleft. The comparative study with fungal ChiJ orthologs identified three regions that display signs of type 1 functional divergence, where unique adaptations in the bacterial and fungal taxa are driven by positive selection.

**Conclusions:**

The identified surface-exposed regions of chitinase ChiJ where sequence diversification is driven by positive selection may putatively be related to functional divergence between bacterial and fungal orthologs. These results show that ChiJ orthologs have evolved under different selective constraints following the horizontal gene transfer event.

## Findings

### Background

Chitin is an insoluble linear *β*-1,4-linked polymer of *N*-acetylglucosamine which is an essential structural component of fungal cell walls. Chitinases (EC 3.2.1.14) are hydrolytic enzymes that catalyse the degradation of chitin and are classified into glycoside hydrolase families 18 and 19, based on amino acid sequence similarity [1,2]. These two families possess different three-dimensional structural folds as well as modes of action. Family 18 chitinases is based on a triosephosphate isomerase (TIM) barrel structure and use a substrate-assisted double-displacement with net retention of the configuration of the anomeric carbon during the catalysis [3].

Biological control of pathogenic fungi causing diseases on important crop plants is an attractive alternative to the extensive use of chemical fungicides in modern agriculture, which may have negative environmental impacts and result in evolution of fungicide resistance in pathogen populations. Certain bacteria from the genus *Streptomyces* and fungi from the genera *Hypocrea* (anamorph *Trichoderma*) and *Bionectria* (anamorph *Clonostachys*) are currently used as biological control agents. The biocontrol ability of these microbes are suggested to be the result of several mechanisms that act synergistically, including direct antagonism by toxins and cell wall degrading enzymes (including chitinases), induced resistance in plants, or competition for nutrients and space [4].

*Streptomyces* bacteria are well known for their production of secreted hydrolytic enzymes and secondary metabolites [5], illustrating their ecological niche as ubiquitous soil bacteria. The genetically best studied species is *S. coelicolor* which is reported to possess 12 different chitinase genes, ten family 18 members (*chiA*, *B*, *C*, *D*, *E*, *H*, *I*, *J*, *K*, *L*) and two family 19 members (*chiF*, *G*) [6]. The ten family 18 chitinases can be phylogenetically divided into subfamilies A, B and C, and subgroups II, IVa and VI [6,7]. Seven genes, *chiA*, *B*, *C*, *D*, *F*, *I* and *J*, are reported to be induced to varying degrees in the presence of colloidal chitin [6,8], while studies of enzymatic activity are reported for *S. coelicolor* ChiA, C, D, F and G [6,9]. Recently, *chiJ* is shown to have an ortholog in *Hypocrea* species (*chi18-15*/*chit36*) and in *Bionectria ochroleuca* (*ech37*), acquired through horizontal gene transfer (HGT) [7,10,11].

Certain *Hypocrea* and *Bionectria* species are mycoparasitic, i.e. they have the ability to parasitize other fungi and use them as a source of nutrients. Studies of gene family evolution show that the mycoparasitic lifestyle has resulted in selection for increased number of cell wall degrading chitinases in *Hypocrea*[10]. In addition, certain *Hypocrea* chitinases evolve rapidly in a manner consistent with co-evolutionary interactions between parasite and host [10], similar to certain plant chitinases involved in defence against fungal pathogens [12,13].

In this study we investigate the molecular evolution of *Streptomyces* chitinase ChiJ, under the hypothesis that its involvement in interactions with soil fungi have resulted in sequence diversification between taxa. In addition, the HGT event presents an opportunity for a comparative investigation of the molecular fate of ChiJ orthologs evolving in bacterial and fungal species, respectively. We hypothesize that bacterial and fungal ChiJ orthologs evolve differently due to the fundamentally different organismal environments.

## Materials and methods

### Identification of genes and phylogenetic analysis

*Streptomyces* genome sequences available at the National Center for Biotechnological Information (NCBI) were screened for the presence of chitinase *chiJ* [GenBank:NP_626743], DNA recombinase A *recA* [GenBank:NP_629894] and DNA polymerase III subunit beta *dnaN* [GenBank:NP_628065] using BLAST [14]. GenBank [15] sequences were screened with ChiJ using BLAST. Sequences were aligned with Clustal X [16]. Phylogenetic analysis was performed using maximum likelihood methods implemented in PhyML-aLRT ver. 2.4.5 [17,18]. The JTT amino acid substitution model [19] was used, the proportion of invariable sites was set to 0, one category of substitution rate was used and gaps were treated as unknown characters. The starting tree to be refined by the maximum likelihood algorithm was a distance-based BIONJ tree estimated by the program [17]. Statistical support for phylogenetic grouping was assessed by approximate likelihood-ratio tests based on a Shimodaira-Hasegawa-like procedure [18] and by bootstrap analysis (1000 resamplings).

### Homology modelling

Similar sequences were located by BLAST in the protein entries of GenBank and aligned using Clustal W [20]. Similar chitinase catalytic module structures (Additional file 1) were obtained from the Protein Data Bank (PDB [21]), then superimposed and compared with the program O [22]. Multiple sequence alignments were used to identify the best pair-wise alignment of the *Streptomyces sp.* Mg1 [GenBank:ZP_04999219] enzyme with that of *Lactococcus lactis* subsp. *lactis*. This pair-wise alignment was the basis of creating a homology model, with PDB entry [PDB:3IAN] (*L. lactis* subsp. *lactis*) as the template in the program SOD [23]. The model was adjusted in O, using rotamers that would improve packing in the interior of the protein. The model is available upon request from the authors. The figure was prepared using O, MOLSCRIPT [24] and Molray [25].

### Reverse conservation analysis

Amino acid diversity in sequence alignments were analysed using Reverse Conservation Analysis (RCA). Amino acid sequences were aligned by Clustal X [16] and amino acid conservation at each position was analysed by RCA analysis as described by Lee (2008) [26]. In short, Rate4Site (Version 2.01) was used to calculate the degree of conservation (S score) for each amino acid position using the empirical Bayesian method [27,28]. A sliding window-average (n = 7) S score was plotted (W mean score) and significant peaks were defined by intensity (I) values of 0.5, as recommended by Lee (2008) [26].

### Codon-based analyses

The rate of non-synonymous (dN) and synonymous (dS) substitutions at each codon, and identification of sites evolving under positive or negative selection, was determined using random effects maximum likelihood models (REL) [29] implemented in the HyPhy software package [30] through the Datamonkey webserver [31]. The optimal nucleotide substitution model was estimated for each gene separately [32] and included the following modifications to the general reversible nucleotide model [29] for *chiJ*: C↔G: R_1_, C↔T: R_AC_, G↔T: R_AT_; for *recA*: A↔C: R_1_, A↔T: R_1_, C↔G: R_CG_, C↔T: R_CT_, G↔T: R_GT_, and for *dnaN*: A↔C: R_1_, A↔T: R_1_, C↔G: R_CG_, G↔T: R_1_. A Bayes factor value ≥ 50 (default) was used as an indication of strong positive selection at a site, while values between 10 and 49 were considered to indicate weak support of positive selection [29].

Identification of co-evolving sites was done using the Spidermonkey/BGM program [33] implemented in the HyPhy software package [30] through the Datamonkey webserver [31]. The same nucleotide substitution models as in REL analysis was used, global dN/dS values were estimated by the program, ambiguous characters were averaged, a two-parent, directed network was used and sites were selected based on non-synonymous branch counts (threshold ≥ 3). A posterior probability value ≥ 0.5 (default) was used as the definition of association between sites.

## Results

### *Identification of* chiJ *orthologs*

**Figure 1:**
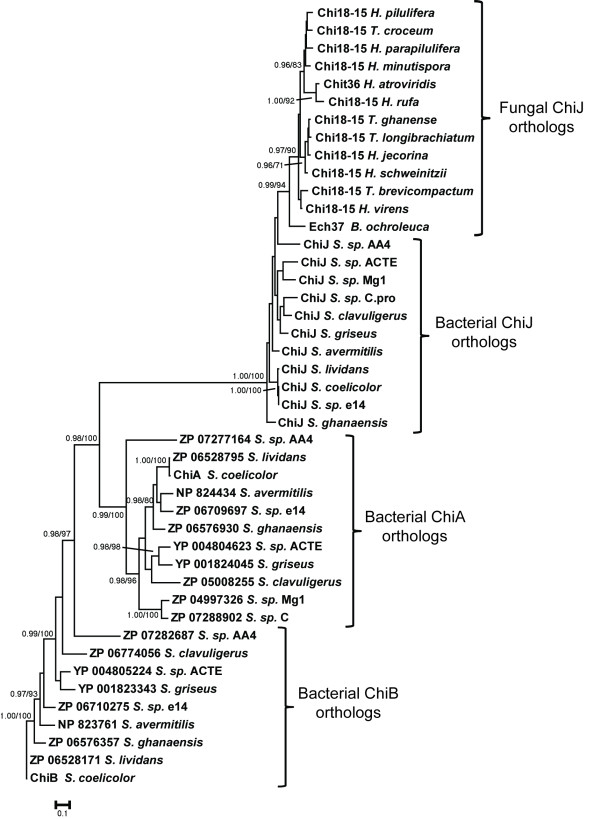
**Phylogenetic relationships of *Streptomyces *ChiJ and related proteins. **Phylogenetic analyses were performed using maximum likelihood methods as implemented in PhyML-aLRT, based on an alignment of chitinase amino acid sequences. Branch support values (approximate likelihood-ratio test probabilities (> 0.95) / bootstrap proportions (> 70%)) are associated with nodes. The bar marker indicate the number of amino acid substitutions per 100 amino acids. *Streptomyces *ChiA, ChiB, ChiJ and fungal ChiJ orthologs are indicated. Sequence identifiers include protein name or locus ID.

The translated *chiJ* gene from *S. coelicolor* was used as the template for screening *Streptomyces* genome sequences available at NCBI. *ChiJ* orthologs were present in ten species, *S. avermitilis*, *S. clavuligerus*, *S. ghanaensis*, *S. griseus*, *S. lividans*, *S. sp*. AA4, *S. sp*. ACTE, *S. sp*. C, *S. sp*. e14 and *S. sp*. Mg1, in addition to *S. coelicolor*. With the exception of *S. sp.* Mg1 and *S. sp.* C that only contained one additional similar gene (E-value ≤ 1e^-5^), the other nine species contained two additional similar genes (E-value ≤ 1e^-5^). A phylogenetic analysis of translated amino acid sequences confirmed that the identified proteins represented the three *Streptomyces* chitinase paralogs ChiA, ChiB and ChiJ in the IVa group [6,7], and confirmed the orthologous status of the identified *chiJ* sequences (Figure 1). The translated *S. coelicolor* ChiJ protein was 358 amino acids long and consisted of a signal peptide for secretion and a family 18 chitinase catalytic module, but no additional substrate-binding modules [34]. *Streptomyces recA* and *dnaN* were present in the selected bacterial genomes, and were retrieved for comparative purposes.

### Homology modelling of ChiJ

**Figure 2:**
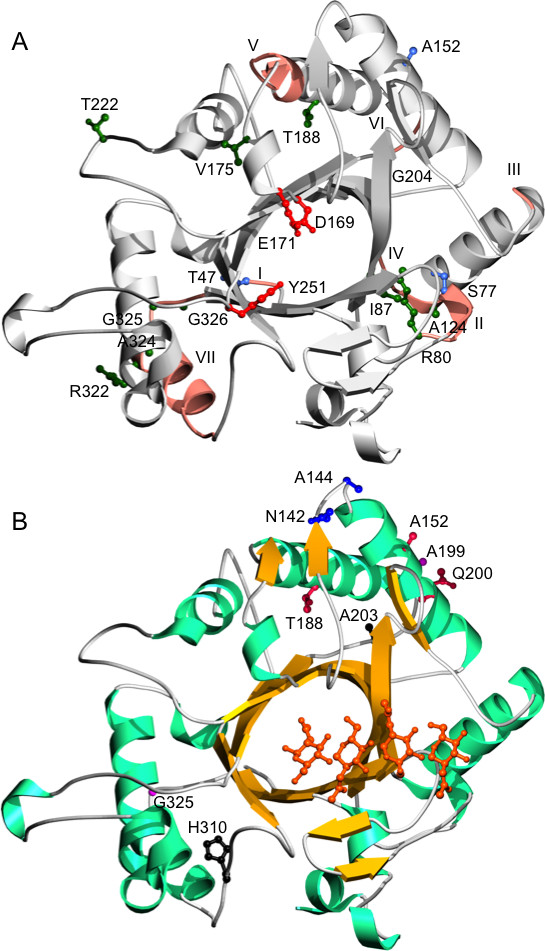
**Homology model of *Streptomyces *sp. Mg1 ChiJ chitinase. A**. Catalytically important residues are marked in red. Amino acids under strong and weak positive selection are shown in royal blue and dark green respectively. Variable regions from reverse conservation analysis are coloured in salmon and marked I-VII (I = 44–47, II = 75–80, III = 110–111, IV = 123–127, V = 178–185, VI = 200–203, VII = 312–327). **B**. Secondary structure elements of the enzyme are coloured differently. Side chains of co-evolving amino acids are shown in balls and sticks. Co-evolving groups of residues are illustrated in the same colour. Chito-tetraose is modelled to show the substrate-binding cleft.

Homology modelling revealed that the *Streptomyces sp.* Mg1 chitinase had a TIM barrel fold (Figure 2) with six insertions and three deletions compared to the chitinase structure from *L. lactis* subsp. *lactis*. In comparison to the hevamine chitinase from rubber tree [35] the catalytic cleft of ChiJ was more open at the substrate entry side of the cleft. Catalytic proton donor (E_171_) and the residues assisting in the catalysis (D_169_ and Y_251_) were conserved. Residues along the cleft that may play important roles in substrate binding were also conserved. The homology model of ChiJ displayed structural similarities to retaining endochitinases [36].

### Distribution of amino acid diversity

Patterns of amino acid diversity can provide important information about the type of selective constraints that act on different parts of an enzyme. In distantly related orthologous sequences only functionally important regions are expected to be conserved between species, while other regions are expected to have a low degree of conservation due to accumulation of neutral mutations. In the case of closely related orthologs, such as ChiJ in the current work, a high degree of conservation is expected for most positions as the limited time since speciation has not allowed sufficient sequence diversification by genetic drift. However, as outlined by Lee (2008) [26], residues important for enzyme properties may be expected to display higher diversity than other positions in closely related orthologs due to selection for modified enzymatic properties between species.

**Figure 3:**
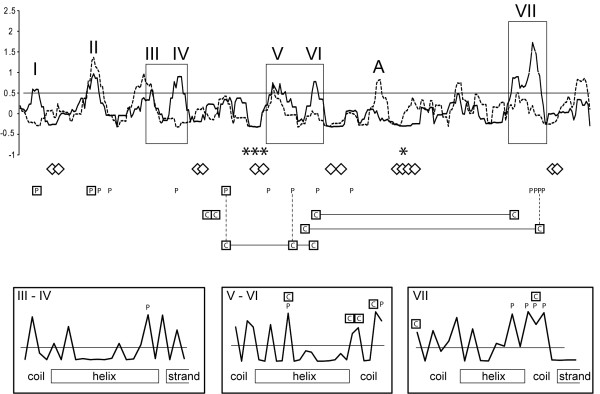
**Reverse conservation analysis of ChiJ orthologs. **Amino acid conservation was estimated using Rate4Site, based on a Clustal X alignment of ChiJ *Streptomyces *orthologs, and plotted as W mean scores in arbitrary units (solid line). Amino acid conservation was estimated in a similar way for fungal orthologs in a previous work [10], and included in the figure (dashed line). Horizontal line indicates a 0.5 standard deviation cut-off. The x-axis represent residue position, asterisks (*) indicate positions of predicted catalytic residues, diamonds (◊) indicate predicted substrate-interacting residues, boxed P indicate residues under strong (Bayes factor ≥ 50) positive selection, P indicate residues under weak (Bayes factor 10–49) positive selection, boxed C interconnected by horizontal lines indicate co-evolving residue groups and vertical dashed lines indicate identical residues. The position of highly variable regions successfully visualised by homology modelling are indicated (I through VII). Magnifications illustrate residue S score distribution of the selected region. The position of a highly variable region in the fungal orthologs is indicated by A.

Therefore, amino acid diversity in a ChiJ alignment (Additional file 2) was analysed by RCA. The first 36 amino acids, including the signal peptide, were excluded from the analysis as this part could not be reliably aligned. High amino acid diversity was distributed amongst seven regions, labelled I through VII, with W mean scores above the 0.5 standard deviation threshold from the RCA analysis (Figure 3). All predicted residues important for catalysis and substrate-binding (Additional file 3) were localised in conserved regions with low W mean scores (Figure 3). Structural positions of the identified regions were visualized on the ribbon cartoon of the homology model of *S. sp.* Mg1 ChiJ (Figure 2A). *S. sp.* Mg1 ChiJ was used as a reference sequence for residue positions throughout this paper. All seven regions with high amino acid diversity (W score ≥ 0.5) were predicted to be surface-exposed, but neither forms a part of the catalytic cleft (Figure 2A). A closer examination of the S scores for individual residue positions revealed that all seven regions of high amino acid diversity were associated with coil regions that connect certain α-helices and β-strands in the family 18 chitinase TIM barrel fold structure (Figure 3). More specifically, highly divergent residue positions (S scores ≥ 0.5) in region I were located in a coil region placed on the N-terminal side of a β-strand at residue positions 50–56. Regions II, III, V, VI and VII contained highly divergent residue positions located in coil regions adjacent to α-helices (pos. 77–79, 111–125, 183–198, 316–323) (Figure 2). Region IV was associated with a α-helix / β-strand junction (Figure 3).

### Evolutionary patterns among sites

High sequence diversity in certain regions and sites between closely related orthologs may be the result of either low selective constraint, i.e. mutations do not alter enzyme function in a significant manner, or to positive selection where certain mutations provide a selective advantage that improve enzyme function. The ratio between non-synonymous and synonymous substitutions is expected to approach 1 in the case of low selective constraints and be > 1 in the case of positive selection.

Therefore, the rate of dN and dS at each codon and identification of sites evolving under positive or negative selection was determined using REL. The first 117 bp of *chiJ* was removed from the analysis as this part could not be reliably aligned. REL fits both dN and dS substitution rates into three discrete rate classes, yielding a total of nine different rate classes of dN/dS. For *chiJ*, one rate class was estimated to have a dN/dS value above 1 (dN (0.37) / dS (0.22) = 1.68). Three sites evolved under strong positive selection (Bayes factor ≥ 50) and an additional eleven sites evolved under weak positive selection (Bayes factor 10 – 49) (Table 1). From these fourteen sites, eleven were associated with coil-regions exhibiting high amino acid diversity from the RCA analysis (Figure 3). In addition, site I_87_ was predicted to be located in a β-strand but not surface-exposed. Both A_152_ and T_222_ were predicted to be surface-exposed but located in a α-helix and a coil region, respectively (Figure 2A).

**Table 1:** Positively selected sites in ChiJ

**Amino acid position**	**Posterior probability**	**Bayes factor**
T_47_	0.77	68
S_77_	0.73	53
R_80_	0.66	39
I_87_	0.34	10
A_124_	0.69	46
A_152_	0.91	197
V_175_	0.68	43
T_188_	0.65	38
G_204_	0.40	13
T_222_	0.63	34
R_322_	0.55	25
A_324_	0.45	16
G_325_	0.61	31
G_326_	0.44	16

Site positions are given in reference to *S. sp.* Mg1 ChiJ. Posterior probabilities and Bayes factors are determined using random effects likelihood method.

For comparative purposes, REL analysis was performed on sequence alignments of two *Streptomyces* genes whose functions were assumed to be independent from chitin degradation or microbial interactions, *recA* and *dnaN*. No rate class was estimated to have a dN/dS value above 1 for either *recA* or *dnaN*.

### Co-evolution between sites

Amino acid residues in an enzyme can interact functionally to perform a specific function, or structurally to stabilize protein structures. Co-evolution between sites can result from selection for mutations that compensate for other, slightly deleterious mutations, to preserve protein function or structure. Identification of co-evolving sites in a protein may thus provide information about the boundaries of functional or structural domains. Functional domains can be especially difficult to define as functionality may depend on residues that are distributed over the whole protein.

Therefore, co-evolution between sites in *chiJ* was studied using evolutionary-network models implemented in Spidermonkey/BGM. Five interacting pairs of sites were identified (Table 2), forming four groups of co-evolving sites (Figure 3). Two co-evolving groups contained residues located in coil-regions VI and VII (Figure 3), located distantly to the catalytic cleft of the homology model of ChiJ (Figure 2A and B). Another group of three co-evolving sites connected region VI with site A_152_, evolving under strong positive selection (Figure 3). Finally, the co-evolving N_142_ – A_144_ pair was predicted to be physically close to site A_152_ and regions V and VI in the homology model (Figure 2A and B). In three cases, co-evolving sites were also evolving under positive selection (Figure 3).

**Table 2:** Co-evolving sites in ChiJ

**Amino acid position 1**	**Amino acid position 2**	**Posterior probability**
N_142_	A_144_	0.85
A_152_	Q_200_	0.59
T_188_	Q_200_	0.55
A_199_	G_325_	0.58
A_203_	H_310_	0.68

Site positions are given in reference to *S. sp.* Mg1 ChiJ. Posterior probabilities for site 1 and site 2 to be conditionally dependent, determined by Spidermonkey/BGM, is given.

### Comparison between chiJ and fungal orthologs

Fungal species of the genera *Hypocrea* and *B. ochroleuca* were previously shown to possess an ortholog to *chiJ* (*chi18-15*/*chit36* and *ech37* respectively), acquired through HGT from its actinobacterial origin [7,10,11]. An alignment of ChiJ (excluding the first 36 amino acids) from *S. coelicolor* and *S. sp.* Mg1, chi18-15 from *H. jecorina*, CHIT36 from *H. atroviridis* and Ech37 from *B. ochroleuca* shows that no insertions or deletions have occurred since the HGT event (data not shown). However, when comparing regions exhibiting high amino acid diversity from the RCA analysis in the current paper with a previous analysis on the fungal (*Hypocrea*) ChiJ orthologs [10], several differences were observed. ChiJ regions I and IV both exhibited high amino acid variation between *Streptomyces* species and contained rapidly evolving sites, while these regions were highly conserved in the fungal counterparts (Figure 3). The same trend was observed for ChiJ regions VI and VII (Figure 3). On the other hand, one region (region A) in the fungal chi18-15/CHIT36 protein alignment was highly variable between fungal species at the amino acid level, but conserved between bacterial ChiJ orthologs (Figure 3).

## Discussion

Adaptive evolution of chitinases have previously been shown for plant chitinases involved in defence against fungal pathogens [12,13], and in fungal chitinases involved in mycoparasitic interactions [10]. The common theme between these examples is that parasite – host interaction seem to exert selection for rapid modification of the involved enzymes in an arms race fashion. Enzyme modification may thus partly account for variation in virulence or disease resistance in the parasite and host, respectively.

The biological function of ChiJ is suggested to include degradation of exogenous chitin for nutrient acquisition and aggressive interactions with soil fungi. Evolution of ChiJ may be influenced by the presence of several additional chitinases in certain *Streptomyces* species [6]. Conservation of gene duplications may be favoured if high levels of a specific chitinase is needed. However, the substantial divergence between ChiA, ChiB and ChiJ that is evident from our phylogenetic analysis suggest that these paralogous proteins represent isozymes with different functional properties that may act synergistically in chitin degradation. Presence of a fungal *chiJ* ortholog in both *Hypocrea spp*. and in *B. ochroleuca* suggests that the HGT event preceded the split of these species. These species belong to the same order (*Hypocreales*) but different families, so the HGT event cannot be considered to be recent. *B. ochroleuca* and certain *Hypocrea* species are mycoparasitic, and thus Ech37/CHIT36 are suggested to participate in degradation of the cell wall of antagonistic fungal species [36,37]. Expression data show that *ech37* in *B. ochroleuca* is indeed induced during interaction with *Botryotinia fuckeliana* on strawberry leaves [37], while *chit36* in *T. asperellum* is induced during plate confrontation with *Rhizoctonia solani*[38].

No region of high amino acid diversity or sites showing signs of adaptive evolution constitute parts of the catalytic cleft of ChiJ. This suggests that adaptation for changes in substrate-specificity between different *Streptomyces* species is not the primary selection force for ChiJ evolution. This conclusion is further supported by the localization of all residues predicted to be important for catalysis and substrate-binding to conserved regions. In addition, *Hypocrea spp.* Chi18-15/CHIT36 interspecific sequence variability is also reported to localize to other parts of the enzyme than the catalytic cleft [10]. This may indicate that the primary substrate of ChiJ and Chi18-15/CHIT36 are of similar composition, presumably fungal cell wall material.

Instead, regions with high amino acid diversity and sites under positive selection in ChiJ coincided with coil regions that connect certain α-helices and β-strands in the family 18 chitinase TIM barrel structure. This result is not surprising as the basic architecture of the TIM barrel is highly conserved between species, while surface loops may vary considerably due to low selective constraints which results in accumulation of neutral mutations. However, some of these surface loops will also be determinants of enzyme properties and interactions with other proteins, and may thus be targets for diversifying selection. Therefore, a major challenge in studies of enzyme evolution is to determine if the observed sequence diversity between orthologs is due to low selective constraints or due to positive selection. Identification of positive selection in a gene strongly implies that these changes are the result from diversifying selection and that the involved sites are connected to protein function.

In the current work, regions I, II, IV, V, VI and VII all contain sites evolving under positive selection. Hence, the most valid interpretation is that the high amino acid diversity between *Streptomyces* species in these regions is the results from diversifying selection and may influence functional properties of ChiJ. The localization of the variable parts to certain coil-regions predicts changes in enzyme architecture between species. Another observation is that two pairs of co-evolving sites are located on opposite sides of the enzyme, indicating that they participate in the same functional domain, i.e. slightly deleterious mutations in the substrate side of ChiJ may be compensated by other mutations on the products side, or vice versa. The surface-exposure of all seven regions of high amino acid diversity may suggest that interactions with environmental factors are the cause of the observed interspecific variability. No single factor can be identified at this stage, but may include interactions with chitinase inhibitors that are produced from soil fungi [39], plants [40], or from *Streptomyces* itself [41]. ChiJ may also interact and evolve together with chitin-binding proteins that enhance the efficiency of the degradation process [42,43]. A comparison of the enzymatic activities of *S. coelicolor* ChiA, ChiC, ChiD and ChiF showed that both pH optima and hydrolytic activity against soluble or crystalline chitin differed substantially between the enzymes [6]. Sites evolving under positive selection in ChiJ are candidates for targeted mutagenesis to elucidate mutational effects on enzyme properties.

The comparison between ChiJ and its fungal orthologs identified two regions that display high amino acid diversity in combination with sites evolving under positive selection in *Streptomyces* but not in fungi (regions I and IV), and one region of high sequence diversity in the fungal orthologs only (region A). This difference in amino acid conservation/diversity patterns is a hallmark of selection for type 1 functional divergence [44]; if the observed diversity in these regions is due to low selective constraints we would expect a similar level of diversity in both bacterial and fungal orthologs. Regions I and IV may thus represent areas on the ChiJ surface that contain structural features that specifically interact with environmental factors, such as inhibitors or chitin-binding helper proteins, present in the ecological niche of *Streptomyces* but not *Hypocrea*. Similarly, region A with high amino acid diversity between fungal orthologs may represent adaptations after the HGT event towards fungal preferences. In fact, this particular region was previously identified in *Hypocrea* chi18-15/CHIT36 to contain sites evolving under positive selection [10]. Furthermore, that study also shows discrete differences in amino acid conservation pattern between *Hypocrea* clades in this region; species in clades *Rufa* and *Pachybasioides* display high interspecific amino acid diversity in this region compared to a conserved interspecific pattern in other clades [10].

In summary, we show that several surface-exposed regions of *Streptomyces* chitinase ChiJ evolves rapidly and that this divergence is driven by selection for adaptive modifications. This shows that ChiJ function, whether it be interactions with soil fungi or nutrient acquisition, is important in the ecological niche of *Streptomyces spp*. We also identify three regions that display signs of type 1 functional divergence, where unique adaptations in the bacterial and fungal taxa are driven by positive selection. These results are noteworthy as it shows that different regions of the ChiJ orthologs have evolved under different selective constraints following the HGT event. The identified regions may thus influence functional properties of ChiJ.

### Availability of supporting data

The data set supporting the results of this article is included within the article (and its additional files).

## Abbreviations

HGT: Horizontal gene transfer; NCBI: National center for biotechnological information; PDB: Protein data bank; RCA: Reverse conservation analysis; REL: Random effects maximum likelihood; TIM: Triosephosphate isomerase.

## Competing interests

The authors declare that they have no competing interests.

## Authors’ contributions

WU carried out homology modelling studies and helped to draft the manuscript. MK conceived of the study, carried out studies of molecular evolution and wrote the manuscript. Both authors read and approved the final manuscript.

## Supplementary Material

Additional file 1 Protein structures used for structural alignment. Click here for file

Additional file 2 **Alignment of *Streptomyces sp.*ChiJ sequences.** Click here for file

Additional file 3 Predicted catalytic and substrate binding residues in ChiJ. Click here for file

## References

[B1] Henrissat B A classification of glycosyl hydrolases based on amino acid sequence similarities Biochem J 1991 280 309 316 1747104 10.1042/bj2800309 PMC1130547

[B2] Henrissat B Bairoch A New families in the classification of glycosyl hydrolases based on amino acid sequence similarities Biochem J 1993 293 781 788 8352747 10.1042/bj2930781 PMC1134435

[B3] Tews I van Scheltinga ACT Perrakis A Wilson KS Dijkstra BW Substrate-assisted catalysis unifies two families of chitinolytic enzymes J Am Chem Soc 1997 119 7954 7959 10.1021/ja970674i

[B4] Harman GE Howell CR Viterbo A Chet I Lorito M Trichoderma species - Opportunistic, avirulent plant symbionts Nature Rev Microbiol 2004 2 43 56 10.1038/nrmicro797 15035008

[B5] Hodgson DA Primary metabolism and its control in Streptomycetes: A most unusual group of bacteria Adv Microb Physiol 2000 42 47 238 10907551 10.1016/s0065-2911(00)42003-5

[B6] Kawase T Yokokawa S Saito A Fujii T Nikaidou N Miyashita K Watanabe T Comparison of enzymatic and antifungal properties between family 18 and 19 chitinases from S. coelicolor A3(2) Biosci Biotech Bioch 2006 70 988 998 10.1271/bbb.70.988 16636468

[B7] Karlsson M Stenlid J Evolution of family 18 glycoside hydrolases: Diversity, domain structures and phylogenetic relationships J Mol Microbiol Biotechnol 2009 16 208 223 10.1159/000151220 18679019

[B8] Saito A Ishizaka M Francisco PB Fujii T Miyashita K Transcriptional co-regulation of five chitinase genes scattered on the Streptomyces coelicolor A3(2) chromosome Microbiol Sgm 2000 146 2937 2946 10.1099/00221287-146-11-2937 11065372

[B9] Heggset EB Hoell IA Kristoffersen M Eijsink VGH Vårum KM Degradation of chitosans with chitinase G from Streptomyces coelicolor A3(2): Production of chito-oligosaccharides and insight into subsite specificities Biomacromolecules 2009 10 892 899 10.1021/bm801418p 19222164

[B10] Ihrmark K Asmail N Ubhayasekera W Melin P Stenlid J Karlsson M Comparative molecular evolution of Trichoderma chitinases in response to mycoparasitic interactions Evol Bioinform 2010 6 1 26 10.4137/ebo.s4198 PMC2865166 20454524

[B11] Mamarabadi M Jensen B Lübeck M Three endochitinase-encoding genes identified in the biocontrol fungus Clonostachys rosea are differentially expressed Curr Genet 2008 54 57 70 10.1007/s00294-008-0199-5 18574585

[B12] Bishop JG Dean AM Mitchell-Olds T Rapid evolution in plant chitinases: Molecular targets of selection in plant-pathogen coevolution P Natl Acad Sci USA 2000 97 5322 5327 10.1073/pnas.97.10.5322 PMC25827 10805791

[B13] Tiffin P Comparative evolutionary histories of chitinase genes in the genus Zea and family Poaceae Genetics 2004 167 1331 1340 10.1534/genetics.104.026856 15280246 PMC1470951

[B14] Altschul SF Madden TL Schäffer AA Zhang J Zhang Z Miller W Lipman DJ Gapped BLAST and PSI-BLAST: a new generation of protein database search programs Nucleic Acids Res 1997 25 3389 3402 10.1093/nar/25.17.3389 9254694 PMC146917

[B15] Benson DA Karsch-Mizrachi I Lipman DJ Ostell J Wheeler DL GenBank Nucleic Acids Res 2008 36 D25 D30 10.1093/nar/gkn320 18073190 PMC2238942

[B16] Larkin MA Blackshields G Brown NP Chenna R McGettigan PA McWilliam H Valentin F Wallace IM Wilm A Lopez R Thompson JD Gibson TJ Higgins DG Clustal W and clustal X version 2.0 Bioinformatics 2007 23 2947 2948 10.1093/bioinformatics/btm404 17846036

[B17] Guindon S Gascuel O A simple, fast, and accurate algorithm to estimate large phylogenies by maximum likelihood Syst Biol 2003 52 696 704 10.1080/10635150390235520 14530136

[B18] Anisimova M Gascuel O Approximate likelihood-ratio test for branches: A fast, accurate, and powerful alternative Syst Biol 2006 55 539 552 10.1080/10635150600755453 16785212

[B19] Jones DT Taylor WR Thornton JM The rapid generation of mutation data matrices from protein sequences Comp Appl Biosci 1992 8 275 282 1633570 10.1093/bioinformatics/8.3.275

[B20] Thompson JD Higgins DG Gibson TJ Clustal-W - Improving the sensitivity of progressive multiple sequence alignment through sequence weighting, position-specific gap penalties and weight matrix choice Nucleic Acids Res 1994 22 4673 4680 10.1093/nar/22.22.4673 7984417 PMC308517

[B21] Berman HM Westbrook J Feng Z Gilliland G Bhat TN Weissig H Shindyalov IN Bourne PE The Protein Data Bank Nucleic Acids Res 2000 28 235 242 10.1093/nar/28.1.235 10592235 PMC102472

[B22] Jones TA Zou JY Cowan SW Kjeldgaard M Improved methods for building protein models in electron-density maps and the location of errors in these models Acta Crystallogr Sec A 1991 47 110 119 10.1107/S0108767390010224 2025413

[B23] Kleywegt GJ Zou JY Kjeldgaard M Jones TA Rossmann MG, Arnold E Around O International tables for crystallography, Vol F Crystallography of biological macromolecules 2001 Kluwer Academic, Dordrecht 353 356 366–367

[B24] Kraulis PJ Molscript - a program to produce both detailed and schematic plots of protein structures J Appl Crystallogr 1991 24 946 950 10.1107/S0021889891004399

[B25] Harris M Jones TA Molray - a web interface between O and the POV-Ray ray tracer Acta Crystallogr Sec D 2001 57 1201 1203 10.1107/s0907444901007697 11468417

[B26] Lee T Reverse conservation analysis reveals the specificity determining residues of cytochrome P450 family 2 (CYP 2) Evol Bioinform 2008 4 7 16 10.4137/ebo.s291 PMC2614186 19204803

[B27] Mayrose I Graur D Ben-Tal N Pupko T Comparison of site-specific rate-inference methods for protein sequences: Empirical Bayesian methods are superior Mol Biol Evol 2004 21 1781 1791 10.1093/molbev/msh194 15201400

[B28] Pupko T Bell R Mayrose I Glaser F Ben-Tal N Rate4Site: an algorithmic tool for the identification of functional regions in proteins by surface mapping of evolutionary determinants within their homologues Bioinformatics 2002 18 S71 S77 10.1093/bioinformatics/18.suppl_1.S71 12169533

[B29] Pond SLK Frost SDW Not so different after all: A comparison of methods for detecting amino acid sites under selection Mol Biol Evol 2005 22 1208 1222 10.1093/molbev/msi105 15703242

[B30] Pond SLK Frost SDW Muse SV HyPhy: hypothesis testing using phylogenies Bioinformatics 2005 21 676 679 10.1093/bioinformatics/bti079 15509596

[B31] Pond SLK Frost SDW Datamonkey: rapid detection of selective pressure on individual sites of codon alignments Bioinformatics 2005 21 2531 2533 10.1093/bioinformatics/bti320 15713735

[B32] Pond SLK Frost SDW A simple hierarchical approach to modeling distributions of substitution rates Mol Biol Evol 2005 22 223 234 15483327 10.1093/molbev/msi009

[B33] Poon A Lewis F Kosakovsky Pond S Frost S An evolutionary-network model reveals stratified interactions in the V3 loop of the HIV-1 envelope PLoS Comp Biol 2007 3 e231 10.1371/journal.pcbi.0030231 PMC2082504 18039027

[B34] Saito A Fujii T Miyashita K Distribution and evolution of chitinase genes in Streptomyces species: Involvement of gene-duplication and domain-deletion Antonie Van Leeuwenhoek 2003 84 7 15 10.1023/A:1024463113606 12906357

[B35] Bokma E Rozeboom HJ Sibbald M Dijkstra BW Beintema JJ Expression and characterization of active site mutants of hevamine, a chitinase from the rubber tree Hevea brasiliensis Eur J Biochem 2002 269 893 901 10.1046/j.0014-2956.2001.02721.x 11846790

[B36] Viterbo A Haran S Friesem D Ramot O Chet I Antifungal activity of a novel endochitinase gene (chit36) from Trichoderma harzianum Rifai TM FEMS Microbiol Lett 2001 200 169 174 10.1111/j.1574-6968.2001.tb10710.x 11425470

[B37] Mamarabadi M Jensen B Jensen DF Lübeck M Real-time RT-PCR expression analysis of chitinase and endoglucanase genes in the three-way interaction between the biocontrol strain Clonostachys rosea IK726, Botrytis cinerea and strawberry FEMS Microbiol Lett 2008 285 101 110 10.1111/j.1574-6968.2008.01228.x 18557783

[B38] Viterbo A Montero M Ramot O Friesem D Monte E Llobell A Chet I Expression regulation of the endochitinase chit36 from Trichoderma asperellum (T. harzianum T-203) Curr Genet 2002 42 114 122 10.1007/s00294-002-0345-4 12478390

[B39] Arai N Shiomi K Yamaguchi Y Masuma R Iwai Y Turberg A Kolbl H Omura S Argadin, a new chitinase inhibitor, produced by Clonostachys sp FO-7314 Chem Pharm Bull 2000 48 1442 1446 10.1248/cpb.48.1442 11045447

[B40] Hurtado-Guerrero R van Aalten DMF Structure of Saccharomyces cerevisiae chitinase 1 and screening-based discovery of potent inhibitors Chem Biol 2007 14 589 599 10.1016/j.chembiol.2007.03.015 17524989

[B41] Sakuda S Isogai A Matsumoto S Suzuki A Search for microbial insect growth-regulators .2. Allosamidin, a novel insect chitinase inhibitor J Antibiot 1987 40 296 300 10.7164/antibiotics.40.296 3570982

[B42] Vaaje-Kolstad G Horn SJ van Aalten DMF Synstad B Eijsink VGH The non-catalytic chitin-binding protein CBP21 from Serratia marcescens is essential for chitin degradation J Biol Chem 2005 280 28492 28497 10.1074/jbc.M504468200 15929981

[B43] Saito A Miyashita K Biukovic G Schrempf H Characteristics of a Streptomyces coelicolor A3(2) extracellular protein targeting chitin and chitosan Appl Environ Microb 2001 67 1268 1273 10.1128/AEM.67.3.1268-1273.2001 PMC92723 11229920

[B44] Cole MF Gaucher EA Exploiting models of molecular evolution to efficiently direct protein engineering J Mol Evol 2011 72 193 203 10.1007/s00239-010-9415-2 21132281 PMC3183505

