# Supplementary material for: Bacterial and fungal chitinase chiJ orthologs evolve under different selective constraints following horizontal gene transfer
Source: BMC Res Notes. 2012 Oct 24;5:581. doi: 10.1186/1756-0500-5-581 (PMC3506478; doi:10.1186/1756-0500-5-581)
Supplement: Additional file 1 — Protein structures used for structural alignment. [file 1756-0500-5-581-S1.pdf]

Additional file 1. Protein structures used for structural alignment.

Listed structures were aligned and superimposed to understand the domain boundaries, substrate binding and catalysis of ChiJ.

Structure	Species	PDB entry
Chitinase B (ChiB)	*Serratia marcescens*	1E6N[1]
Chitinase B (ChiB)	*Serratia marcescens*	1H0G[2]
Chitinase B (ChiB)	*Serratia marcescens*	1O6I[3]
Lectin (Ym1)	*Mus musculus*	1E9L[4]
Chitinase A (ChiA)	*Serratia marcescens*	1FFR[5]
Hevamine	*Hevea brasiliensis*	1HVQ[6]
Hevamine	*Hevea brasiliensis*	1KQY[7]
Hevamine	*Hevea brasiliensis*	1KQZ[7]
Hevamine	*Hevea brasiliensis*	1KR1[7]
Chitinase A1	*Bacillus circulans*	1ITX[8]
Chitinase	*Aspergillus fumigatus*	1W9P[9]
Haementhin	*Scadoxus multiflorus*	3D5H[10]
Chitinase A (ChiA)	*Streptomyces coelicolor*	3EBV[10]
Chitinase	*Lactococcus lactis subsp. Lactis*	3IAN[10]

[1]van Aalten DM, Komander D, Synstad B, Gåseidnes S, Peter MG, Eijsink VG: **Structural insights into the catalytic mechanism of a family 18 exo-chitinase.** *Proc Natl Acad Sci USA* 2001, **98**:8979-8984.

[2]Houston DR, Shiomi K, Arai N, Omura S, Peter MG, Turberg A, Synstad B, Eijsink VG, van Aalten DM: **High-resolution structures of a chitinase complexed with natural product cyclopentapeptide inhibitors: mimicry of carbohydrate substrate.** *Proc Natl Acad Sci USA* 2002, **99**:9127-9132.

[3]Houston DR, Eggleston I, Synstad B, Eijsink VG, van Aalten DM: **The cyclic dipeptide CI-4 [cyclo-(l-Arg-d-Pro)] inhibits family 18 chitinases by structural mimicry of a reaction intermediate.** *Biochem J* 2002, **368**:23-27.

[4]Sun YJ, Chang NC, Hung SI, Chang AC, Chou CC, Hsiao CD: **The crystal structure of a novel mammalian lectin, Ym1, suggests a saccharide binding site.** *J Biol Chem* 2001, **276**:17507-17514.

[5]Papanikolau Y, Prag G, Tavlas G, Vorgias CE, Oppenheim AB, Petratos K: **High resolution structural analyses of mutant chitinase A complexes with substrates provide new insight into the mechanism of catalysis.** *Biochemistry* 2001, **40**:11338-11343.

[6]Terwisscha van Scheltinga AC, Kalk KH, Beintema JJ, Dijkstra BW: **Crystal structures of hevamine, a plant defence protein with chitinase and lysozyme activity, and its complex with an inhibitor.** *Structure* 1994, **2**:1181-1189.

[7]Bokma E, Rozeboom HJ, Sibbald M, Dijkstra BW, Beintema JJ: **Expression and characterization of active site mutants of hevamine, a chitinase from the rubber tree *Hevea brasiliensis*.** *Eur J Biochem* 2002, **269**:893-901.

[8]Matsumoto T, Nonaka T, Hashimoto M, Watanabe T, Mitsui Y: **Three-dimensional structure of the catalytic domain of chitinase A1 from *Bacillus circulans* WL-12 at a very high resolution.** *Proc Jpn Acad Ser B* 1999, **75**:269-274.

[9]Rao FV, Houston DR, Boot RG, Aerts JM, Hodkinson M, Adams DJ, Shiomi K, Omura S, van Aalten DM: **Specificity and affinity of natural product cyclopentapeptide inhibitors against *A. fumigatus*, human, and bacterial chitinases.** *Chem Biol* 2005, **12**:65-76.

[10]Unpublished.
