# Supplementary material for: Bacterial and fungal chitinase chiJ orthologs evolve under different selective constraints following horizontal gene transfer
Source: BMC Res Notes. 2012 Oct 24;5:581. doi: 10.1186/1756-0500-5-581 (PMC3506478; doi:10.1186/1756-0500-5-581)
Supplement: Additional file 2 — Alignment of Streptomyces sp.ChiJ sequences. [file 1756-0500-5-581-S2.pdf]

Additional file 2. Alignment of *Streptomyces spp.* ChiJ sequences, where a dot indicate an identical amino acid residue as in S. clavuligerus ChiJ and a dash indicate a gap.

```
                            I                     II
                            10    ◊  20 ◊        30        40        50        60        70
                               P                      P  P      P
ChiJ S. clavuligerus  AETCAVKSKPAGKVLQGYWENWDGAANGVHPPLGWIPITDPRIRAHGYNVLNAAFPVIRSDGTVLWENGM
ChiJ S. griseus       V.........T.............................S..TQ.....I................D..
ChiJ S. sp. C         PD.....P.............................V..G.........V................D.F
ChiJ S. sp. ACTE      .D.....PR.Q.....................F..TS.A..L.P......V................D..
ChiJ S. sp. Mg1       .D..P..PR.T...............A.....F..T....S...D.....I.......L....A..QD..
ChiJ S. coelicolor    .D..............................F..T.....Q.G..............L....A...D..
ChiJ S. sp. e14       .D..............................F..T.....Q.G..............L....A...D..
ChiJ S. lividans      .D..............................F..T.V...Q.G..............L....A...D..
ChiJ S. ghanaensis    .D...L.P................SS......F..T....S..AQ.....I............A......
ChiJ S. avermitilis   .D......R.S.............SS......F..T....S..A......I............A...D..
ChiJ S. sp. AA4       SD.....PR.S.P............S.....GM..V..N..S.A......I.......L.....Q..D..

                       III         IV        ◊◊                 C                ◊ ◊
                                80   P    90        100   C C  110   P   120      130 * * *  P 140
ChiJ S. clavuligerus  DTTVKVPTPAEMCQAKADGQTILMSIGGAAAGIDLSSRAVADRFIATIVPILKRFNFDGIDIDIETGLVG
ChiJ S. griseus       .N....A..........S.L.T...............S......VQ.......KY...............
ChiJ S. sp. C         .S..............Q..L.V.............N.T......V.....L..KY...............
ChiJ S. sp. ACTE      .A..R............S.L.V.L.....T.....N.T......VE.V.....AN.............A.
ChiJ S. sp. Mg1       .A..E.A..........A.A...............N.A......V..L.....KY...............
ChiJ S. coelicolor    .RG...A......A...S...L.L.....T.....N.T......VD.......EY.............T.
ChiJ S. sp. e14       .RG...A......A...S...L.L.....T.....N.T......VD.......EY.............T.
ChiJ S. lividans      .RG...A......A...S...L.L.....T.....N.T......VD.......EY.............T.
ChiJ S. ghanaensis    ..G...A......A...A.....L.....T.....N.T.......E.......TY.............I.
ChiJ S. avermitilis   ..G...A..........S.....L.............S......V.....L..KY...............
ChiJ S. sp. AA4       .S....S......A..DV.A.................SS...K.VS.......KY.............SS

                          V        C         VI
                                150 P     160 CC  CP 170    ◊    ◊180    P   190       200       210
ChiJ S. clavuligerus  TGNINQLSASQANLIHIIDGVLAQMPPNFGLTMAPETAYVTGGSVVYGSIWGSYLPIIKKYADNGRLWWL
ChiJ S. griseus       S..........S...R.......A..............................................
ChiJ S. sp. C         S.S.TR..P......R..........AG.................A......A...VV............
ChiJ S. sp. ACTE      S.D.G...P..S...R.......A...G........................A...V.............
ChiJ S. sp. Mg1       S.T.TR..P..T...R....I.....AG.................T......A...V..R..........
ChiJ S. coelicolor    S..........S...R.......R..S.................I.......A.........E.......
ChiJ S. sp. e14       S..........S...R.......R..S.................I.......A.........E.......
ChiJ S. lividans      S..........S...R.......R..S.................I.......A.M...............
ChiJ S. ghanaensis    ........P..T...R......KR..DG........................A.................
ChiJ S. avermitilis   S...G..........R......G...S..................A......A....V............
ChiJ S. sp. AA4       S.D..S........ER......S...SG.................T......A............Q....

                          ◊                                                                VII
                        ◊ * ◊  220 ◊     230       240       250       260       270   C   280
ChiJ S. clavuligerus  NMQYYNGSMYGCSGDSYAAGTVAGFRAQTDCLNRGLVIQGTTIRVPYDKQAPGLPAQPGAGGGHMAPGLV
ChiJ S. griseus       .................S.......T.......K.........K......V..............ST...
ChiJ S. sp. C         .................S.......K......D....V....V.......V............Y......
ChiJ S. sp. ACTE      .................E....Q..T......DK...V....VK......V..............S.AQ.
ChiJ S. sp. Mg1       ......................Q..T.......Q................V..............S.A..
ChiJ S. coelicolor    ............A....S....Q..T......DK........V.......V............Y.S.SP.
ChiJ S. sp. e14       ............A....S....Q..T......DK........V.......V............Y.S.SP.
ChiJ S. lividans      ............A....S....Q..T......DK........V.......V............Y.S.S..
ChiJ S. ghanaensis    .................P....Q..TV......K................V................SQ.
ChiJ S. avermitilis   .................S....Q..T.......K...V....V.......V......S......Y.S.S..
ChiJ S. sp. AA4       .................E....Q..TV..Q..DK..TV........V...V............Y.D..S.

                       VII         C
                                 290       300       310
                          P  PPP       ◊◊
ChiJ S. clavuligerus  TQAWNGYGNALKGLMTWSLNWDGSRNWTFGDNVRALQGR
ChiJ S. griseus       S....H.KGS...............G.......KS....
ChiJ S. sp. C         A....P..GR.............................
ChiJ S. sp. ACTE      A....T.SG-........I......D.......KN....
ChiJ S. sp. Mg1       A....R.AGG........V....A......N..KT....
ChiJ S. coelicolor    S....H.NG...............K.....T..KS....
ChiJ S. sp. e14       S....H.NG...............K.....T..KS....
ChiJ S. lividans      S....H.NG...............K.....T..KS....
ChiJ S. ghanaensis    A....H..TS........I.....K........K.....
ChiJ S. avermitilis   S...RH..SG........I.....K........K.....
ChiJ S. sp. AA4       S..YQSVPG-.....D..I......G.......KS....
```

As outlined in Fig. 3, asterisks (*) indicate positions of predicted catalytic residues, diamonds (◊) indicate predicted substrate-interacting residues, P indicate residues under positive selection, C indicate co-evolving residues. Positions of highly variable regions are indicated (I through VII).
