# Supplementary material for: Bacterial and fungal chitinase chiJ orthologs evolve under different selective constraints following horizontal gene transfer
Source: BMC Res Notes. 2012 Oct 24;5:581. doi: 10.1186/1756-0500-5-581 (PMC3506478; doi:10.1186/1756-0500-5-581)
Supplement: Additional file 3 — Predicted catalytic and substrate binding residues in ChiJ. [file 1756-0500-5-581-S3.pdf]

Additional file 3. Predicted catalytic and substrate binding residues in ChiJ.

Comparison of residues of *Streptomyces* ChiJ model (STRP) corresponding to catalysis (red) and substrate binding (black) in *Serratia marcescens* chitinase A structure [PDB:1FFR] and hevamine [PDB:1HVQ].

	1FFR	1HVQ	STRP
Active site	D313	D125	D169
	E315	E127	E171
	F/Y390	Y183	Y251
	D311	D123	D167
Substrate binding subsites			
-5	Y170	-	-
-4	R172	-	-
-3	W167	-	W58
	T276	I82	A136
	E473	-	-
-2	W275	G81	A135
	T276	I82	A136
	E473	-	-
	W539	W255	W333
	E540	S256	S334
-1	Y163	Y6	Y54
	W275	G81	A135
	D313	D125	D169
	E315	E127	E171
	A362	A156	A210
	M388	Q181	Q249
	F/Y390	Y183	Y251
	D391	N184	N252
	Y444	-	-
	R446	-	-
	W539	W255	W333
+1	W275	G81	A135
	E315	E127	E171
	F316	-	-
	M388	Q181	Q249
	D391	N184	N252
	R446	-	-
+2	W275	G81	A135
	K369	-	Y215
	D391	N184	N252
	F396	-	-
	Y418	-	Y256
